# Neighborhood-Level Stressors and Individual-Level Cardiovascular Disease Risk in Native Hawaiians: a Cross-Sectional Study

**DOI:** 10.5888/pcd21.220341

**Published:** 2024-02-15

**Authors:** Claire Townsend Ing, Hyeong Jun Ahn, Mapuana C.K. Antonio, Adrienne Y. Dillard, Bridget Puni Kekauoha, Kevin Cassel, Scott Abrigo, Michelle Kauhane, Melody S. Halzel, Joseph Keaweʻaimoku Kaholokula

**Affiliations:** 1 Department of Native Hawaiian Health, University of Hawaiʻi at Mānoa, Honolulu, Hawaiʻi; 2 Department of Quantitative Health Sciences, University of Hawai‘i at Mānoa, Honolulu, Hawaiʻi; 3 Native Hawaiian and Indigenous Health, Office of Public Health Studies, University of Hawaiʻi at Mānoa, Honolulu, Hawaiʻi; 4 Kula no na Po‘e Hawai‘i, Honolulu, Hawaiʻi; 5 University of Hawai‘i Cancer Center, University of Hawaiʻi at Mānoa, Honolulu, Hawaiʻi; 6 Kapolei Community Development Corporation, Kapolei, Hawaiʻi

## Abstract

**Introduction:**

Native Hawaiian people have higher rates of illness and death related to cardiovascular disease (CVD) than non-Hispanic White people. Research in other populations has shown that individual-level CVD risk factors (ie, high-fat diet, physical inactivity, obesity, and tobacco use) are associated with neighborhood characteristics (ie, social cohesion, walkability, availability of healthy food, and safety). This association has yet to be examined among Native Hawaiians.

**Methods:**

We conducted a cross-sectional survey of community-dwelling Native Hawaiian people in 2020. Three multiple regression models and 1 logistic regression model were assessed. Each model included individual-level CVD risk factors, age, sex, education, income, and neighborhood characteristics.

**Results:**

The regression models for body mass index (BMI) and physical activity showed significant results. The BMI model (*R* ^2^ = 0.22, *F* = 4.81, *P* < .001) demonstrated that age, sex, education level, physical activity, and percentage of fat in the diet were significantly related to BMI. The availability of healthy foods had a significant, independent relationship with BMI (standardized β = −1.47, SE = 0.53, *P* = .01). The physical activity model (*R* ^2^ = 0.21, *F* = 4.46, *P* < .001) demonstrated that age, sex, education, and BMI were significantly related to physical activity. None of the neighborhood characteristics had significant, independent relationships to physical activity.

**Conclusions:**

We found that neighborhood-level factors improved the model’s ability to explain variance in BMI. Efforts to decrease BMI would benefit from improving the availability of healthy foods in neighborhoods, a finding supported by research in other populations.

**Summary**

**What is already known on this topic?**

Native Hawaiian populations are disproportionately affected by cardiovascular disease. Research in other populations has shown neighborhood-level factors, such as social cohesion, safety, walkability, and availability of healthy foods, can influence individual-level risk factors for cardiovascular disease.

**What is added by this report?**

This study included Native Hawaiians, a population that experiences multiple health disparities but with limited research, in the novel context of Hawaiian Homestead communities. After controlling for individual-level factors, neighborhood factors explained a significant proportion of the variance in body mass index, with availability of healthy food being of particular importance.

**What are the implications for public health practice?**

Efforts to decrease the body mass index among Native Hawaiian homesteaders may benefit from improving the availability of healthy food in neighborhoods.

## Introduction

Native Hawaiians, the Indigenous people of Hawaiʻi, have an age-adjusted prevalence of coronary artery disease (3.0%), heart attack (3.3%), and stroke (3.6%) higher than non-Hispanic White people (2.1%, 1.8%, and 1.6%, respectively) (1–3). Not surprisingly, Native Hawaiian people have high rates of individual-level cardiovascular disease (CVD) risk factors. Compared with non-Hispanic White people, Native Hawaiian people are almost twice as likely to have obesity, less likely to report leisure-time physical activity, and less likely to report 150 minutes of physical activity per week (4–6). Native Hawaiian people are more likely to smoke cigarettes, consume more calories daily, and have poorer diet quality than other ethnic groups (7–9).

In other populations, neighborhood-level factors, such as walkability, access to healthy foods, perceived safety, and social cohesion, have been associated with individual-level CVD risk factors (10–13). For instance, in a national study of US men and women aged 50 years or older (N = 9,032), more positive perceptions of neighborhood social cohesion were associated with higher levels of physical activity (14). Despite the evidence suggesting that neighborhood-level factors are related to individual-level CVD factors, research examining this relationship in Native Hawaiian communities is lacking. The purpose of this study was to examine the relationship of neighborhood factors (ie, social cohesion, walkability, availability of healthy food, and safety) on 4 known individual-level CVD risk factors (ie, percentage of fat in the diet, physical activity frequency, body mass index [BMI], and tobacco use) after controlling for sociodemographic characteristics and the other individual-level risk factors.

## Methods

### Research design

We initiated the Hawaiian Homestead Health Survey Project in 2015 in partnership with 3 Hawaiian homesteads representing 1 community on the island of O‘ahu. We employed a community-based participatory research approach in which community-based and academic-based researchers collaborated to create and administer the survey and analyze and interpret the results. Originally created in 2015, the survey was revised in 2019 to include questions on neighborhood-level stressors (15). The survey includes questions on demographic characteristics, health behaviors, and neighborhood characteristics. The project and its survey were reviewed and approved by the Institutional Review Board and Human Studies Program at the University of Hawai‘i at Mānoa under the title Homestead Health Survey, protocol 22690.

### Procedures

The Hawaiian Homes Commission Act of 1920 set aside over 200,000 acres of Crown and government lands of the Hawaiian Kingdom for Hawaiians with a blood quantum of 50% or higher (16). The lands are located across the state with many in remote and lower-income areas. These government-protected lands can be leased by eligible Native Hawaiians for up to 99 years for $1 a year (17), which can be renewed. As of 2021, approximately 10,000 beneficiaries had homestead leases, for a total of approximately 38,800 people living on homestead lands (18,19).

We mailed the Homestead Health Survey to all 1,000 households in 4 homestead communities on the island of Oʻahu between February and April of 2020. Before mailing the surveys, we provided information about the survey and its intent to community members who attend homestead association meetings. The surveys, as well as a personalized cover letter that described the informed consent process and purpose of the study, were mailed to the homestead lessees; however, any adult household member could complete the survey. The response rate was 30% (300 of 1,000 households).

The survey took approximately 45 minutes to complete and consisted of 5 main sections: 1) Demographics, 2) General Health, 3) Cancer Screening, 4) Health-Related Factors, and 5) Social Relations. Demographic variables included questions related to age, income, employment status, and sex. The General Health section related to personal and family health and included questions about health conditions, trust in health care workers, and the health status of family members. The Cancer Screening section asked about screening for different cancers (ie, breast, cervical, prostate, and colorectal). The Health-Related Factors section asked about individuals’ views on things that may affect their health, such as the support available, life satisfaction, and solving problems. The Social Relations section included questions about acculturation, discrimination, and neighborhood-level stressors.

We provided participants with pre-addressed and stamped return envelopes and asked them to return the survey within 3 weeks of receipt. We mailed reminder postcards approximately 12 and 24 days after the initial mailing to households from which a response had not been received. We provided a $15 gift card to participants who returned their survey as compensation for their time.

### Instruments and measures

#### Demographic variables

We collected the demographic variables age (in years), sex (male or female), level of education, and annual household income. These questions used the same language as items included in the Behavioral Risk Factor Surveillance System (20). We asked participants to select the highest grade or year of school they had completed (ie, less than high school graduate, high school graduate or General Educational Development certificate, some college or technical school, or a college degree). Participants reported their annual household income level based on the range that best represented their current status (ie, <$30,000, $30,000 to <$50,000, $50,000 to <$75,000, or ≥$75,000).

#### Neighborhood level stressors scale

We used the Neighborhood Level Stressor scale to assess participants’ perceptions of their neighborhoods (21). We asked participants to consider 4 neighborhood dimensions within a 1-mile radius of their homes: walkability (7 items), availability of healthy foods (3 items), perceived safety (3 items), and social cohesion (4 items). We defined walkability as how conducive an area is to walking (eg, “It is pleasant to walk in my neighborhood”). Availability of healthy foods was defined as the presence of low-fat and nonfat food options (eg, “A large selection of low-fat products is available in my neighborhood”). Perceived safety was defined as participants’ level of comfort and perception of risk in the neighborhood (eg, “Violence is not a problem in my neighborhood”). Social cohesion was defined as the strength of relationships and solidarity in the community (eg, “People in my neighborhood can be trusted”). Participants indicated how strongly they agreed or disagreed with each statement on a Likert-type scale, ranging from 5 (strongly disagree) to 1 (strongly agree). We computed dimension scores as the mean for all items assessing a specific dimension; lower scores indicated a more positive perception of the relevant factor.

#### Individual cardiovascular disease risk factors

The Homestead Health Survey also asked for weight, height, physical activity frequency, percentage of fat in the diet, and tobacco use. By using self-reported weight in pounds and height in feet and inches, we calculated BMI using the standard calculation (ie, [weight in pounds × 703] ÷ [height in inches^2^]).

We assessed physical activity frequency with multiple-choice questions adapted from the Physical Activity Questionnaire (22). Participants indicated how often they had taken part in moderate or vigorous physical activity on a 5-point scale ranging from 1 (more than 4 times a week) to 5 (rarely or never). We then calculated the mean score, with possible scores ranging from 1 to 5. Higher scores indicated less physical activity.

We assessed the percentage of fat in the diet by using the Percentage Energy from Fat Screener from the National Institutes of Health (23). Participants answered questions about types of food eaten over the past 12 months. Food types included cheese, eggs cooked in butter, fried potatoes, margarine or butter on various foods, and regular-fat mayonnaise or salad dressing. Frequency was assessed on a scale ranging from never to 2 or more times per day. We calculated percentage energy from fat by applying regression coefficients to the frequency of consumption for each food item (24).

We assessed tobacco use by having participants indicate whether they had smoked at least 100 cigarettes in their lifetime. Those who answered yes were considered “ever smokers” and those who answered no were considered “never smokers” (25).

### Data reduction and statistical analysis

We summarized categorical variables by using frequencies. We summarized continuous variables by using means and standard deviations. We imputed missing data such that if a participant had 1 data point missing from a multi-item scale (eg, walkability), it was replaced with the participant’s average score for that factor based on the items answered. Missing data varied by item or scale. Percentage fat in diet had the highest amount missing, 46 of 300. All the other variables had no more than 14 missing (ie, walking and healthy foods) and several had no missing data at all (ie, sex, education, income, physical activity). There was no significant difference in demographic characteristics between those with and without missing data.

We examined the correlations between all variables by using bivariate correlation analyses. We conduced point-biserial correlations to identify neighborhood-level factors significantly associated with the 4 individual-level CVD risk factors of interest (ie, BMI, percentage fat in the diet, physical activity, and tobacco use). To examine the unique contribution of neighborhood-level factors in the explanation of BMI, physical activity, and percentage fat in diet, we performed a hierarchical multiple regression analysis. To examine the unique relationship of neighborhood-level factors to smoking status, we conducted a logistic regression. Each model included the sociodemographic variables of age, sex, education level, and income, the 3 applicable individual-level CVD risk factors, and the 4 neighborhood-level characteristics. The standardized β, standard error of β, and *R* ^2^ are presented for the linear regression models. The odds ratio (OR), 95% CI, and *P* value are presented for the logistic regression model.

## Results

### Participant characteristics

Two-thirds of the 300 participants who responded were women (66.3%) and the mean (SD) age was 53.7 (14.7) (Table 1). The education level of the participants was fairly equally distributed across 3 categories: college graduates (32.6%), some college (29.0%), and high school diploma (32.6%). Most participants (41.6%) had an annual household income of $75,000 or more, with the remaining participants evenly distributed in the other income categories. The mean (SD) BMI was 31.3 (7.4) and participants got 33.8% of the calories in the diet from fat.

**Table 1:** Characteristics of Participants (N = 300) in the Hawaiian Homestead Health Survey, 2020

Characteristics	Mean (SD) or N (%)^a^
Female	199 (66.3%)
Age, y	53.7 (14.7)
Education level	
Less than high school graduate	16 (5.3%)
High school graduate or General Educational Development certificate	98 (32.6%)
Some college or technical school	87 (29.0%)
College degree	98 (32.6%)
Annual income, $	
<30,000	53 (17.6%)
30,000 to <50,000	46 (15.3%)
50,000 to <75,000	53 (17.6%)
≥75,000	125 (41.6%)
Body mass index^b^	31.3 (7.4)
Ever smoker^c^	105 (35.1%)
Physical activity level^d^	3.1 (1.4)
Percentage of fat in diet^e^	33.8 (6.8)
Walkability^f^	2.2 (0.7)
Availability of healthy food^f^	2.6 (1.0)
Safety^f^	2.8 (0.9)
Social cohesion^f^	3.1 (1.0)

a Percentages may not add up to 100 because of missing data.

b Calculated as (weight in pounds × 703) ÷ (height in inches^2^).

c Participants who said that they had smoked at least 100 cigarettes in their lifetime.

d Calculated based on responses to multiple-choice questions adapted from the Physical Activity Questionnaire (22). Responses were in a range from 1 (more than 4 times a week) to 5 (rarely or never).

e Assessed by using the Percentage Energy from Fat Screener from the National Institutes of Health (23). Frequency of eating certain food types was assessed on a scale ranging from never to 2 or more times per day. We calculated percentage energy from fat by applying regression coefficients to the frequency of consumption for each food item (24).

f The Neighborhood Level Stressor scale (21) was used for participants to indicate how strongly they agreed or disagreed with statements on a Likert-type scale, ranging from 5 (strongly disagree) to 1 (strongly agree). Walkability was defined as how conducive an area is to walking. Availability of healthy foods was defined as the presence of low- and non-fat food options. Perceived safety was defined as participants’ level of comfort and perception of risk in the neighborhood. Social cohesion was defined as the strength of relationships and solidarity in the community.

For neighborhood-level factors, the mean (SD) scores for walkability (2.2 [0.7]), availability of healthy food (2.6 [1.0]), and safety (2.8 [0.9]) were all lower than 3, indicating that participants perceived their neighborhoods as having low walkability, low safety, and low availability of healthy foods. For all the neighborhood-level factors measured, participants had the most favorable perception (indicated by the lower relative score) of the neighborhood’s walkability compared with the other neighborhood-level factors. Participants had the least favorable perception of social cohesion, indicated by the highest relative score (3.1 [1.0]).

### Bivariate analysis

Of the individual CVD risk factors (ie, percentage of fat in the diet, physical activity frequency, BMI, and tobacco use), only BMI was significantly correlated with the neighborhood-level factors of availability of healthy food (*r* = 0.119, *P* = .05), safety (*r* = 0.14, *P* < .02), and social cohesion (*r* = 0.14, *P* = .02) (Table 2). This indicates that when the neighborhood is perceived to be safe, to have a high level of social cohesion, and to have healthy foods available, residents have a lower BMI. Additionally, BMI had significant bivariate correlations with sex (*r* = −0.13, *P* < .03) and education level (*r* = −0.15, *P* = .01), indicating that male participants had significantly higher BMIs than female participants and that higher levels of education were associated with lower BMIs.

**Table 2:** Intercorrelation Matrix of Cardiovascular Disease Risk Factors and Participant Characteristics in the Hawaiian Homestead Health Survey, 2020

Variable^a^	Age	Edu	Income	BMI	ES	PA	PFD	Walk	AHF	Safety	SC
Sex	−0.14^b^	0.11^b^	−0.05	−0.13^b^	−0.05	−0.02	0.05	0.06	0.01	0.12	0.01
Age	—	−0.10	−0.04	−0.05	0.10	0.09	−0.18^c^	−0.09	−0.19^c^	−0.03	−0.02
Education level		—	−0.06	−0.15^b^	0.04	−0.03	0.03	0.05	−0.03	0.16^c^	−0.05
Income			—	0.10	−0.05	0.11^b^	0.11	0.02	0.08	−0.01	0.07
Body mass index				—	−0.01	0.01	0.09	0.08	0.12^b^	0.14^b^	0.14^b^
Ever smoker					—	0.20^c^	−0.04	0.08	−0.01	−0.04	0.02
Physical activity						—	−0.01	−0.01	−0.05	0.04	0.09
Percentage of fat in diet							—	0.00	0.05	−0.08	−0.04
Walkability								—	0.48^c^	0.28^d^	0.25^d^
Availability of healthy foods									—	0.16^c^	0.17^c^
Safety										—	0.29^d^
Social cohesion											—

Abbreviations: AHF, availability of healthy foods; BMI, body mass index; Edu, education level; ES, ever smoker; PA, physical activity level; PFD, percentage of fat in diet; SC, social cohesion; Walk, walkability.

a Details about the variables are explained in footnotes to Table 1.

b *P* < .05.

c *P* < .01.

d *P* < .001.

### Regression analysis

Three hierarchical regression models (Table 3; physical activity, percentage fat in diet, and BMI) and 1 logistic regression model (Table 4; smoking status) were conducted to examine the relationship between neighborhood-level factors and individual-level CVD risk factors, controlling for sociodemographics. The models on percentage fat in diet and smoking status did not yield significant results (*R* ^2^ = 0.07, *F* = 1.32, *P* = .20 for percentage of fat in diet; χ^2^ = 4.80, *P* = .78 for smoking status).

**Table 3:** Hierarchical Regression Analyses of Individual-Level and Neighborhood-Level Factors Associated With Cardiovascular Disease Risk Factors (n = 234) in the Hawaiian Homestead Health Survey, 2020

Variable^a^	Model physical activity			Model percentage of fat in diet			Model body mass index		
	β	SE β	*P* value	β	SE β	*P* value	β	SE β	*P* value
**Sociodemographic factors**									
Sex	−1.06	0.19	<.01	−2.06	1.05	.05	3.83	1.02	<.01
Age	0.02	0.01	<.01	−0.05	0.03	.16	−0.07	0.03	.04
Education level^b^									
Less than high school graduate	−0.51	0.44	.25	−0.98	2.35	.68	7.12	2.30	.04
High school graduate or General Educational Development certificate	−1.06	0.45	.02	−2.16	2.44	.38	8.28	2.37	<.01
Some college or technical school	−0.75	0.44	.09	−1.59	2.37	.50	5.83	2.34	.01
Income	−0.00	0.00	.60	0.01	0.02	.75	0.03	0.02	.11
**Individual-level factors**									
Physical activity	—	—	—	−0.47	0.36	.19	1.48	0.34	<.01
Percentage of fat in diet	−0.02	0.01	.19	—	—	—	0.14	0.07	.03
Body mass index	0.05	0.01	<.01	0.14	0.07	.03	—	—	—
Ever smoker	0.01	0.01	.39	−0.02	0.03	.61	−0.05	0.03	.15
**Neighborhood-level factors**									
Walkability	0.25	0.15	.10	0.19	0.82	.82	−1.08	0.81	.19
Availability of healthy foods	−0.03	0.10	.75	0.12	0.54	.82	1.47	0.53	<.01
Safety	−0.06	0.12	.60	0.10	0.64	.88	0.54	0.64	.40
Social cohesion	−0.06	0.11	.56	−1.15	0.57	.04	0.61	0.57	.28
*R^2^*		0.21			0.07			0.22	
*F* value		4.46			1.32			4.81	
*P* value		<.001			.20			<.001	

a Details about the variables are explained in footnotes to Table 1.

b Education reference group is college graduate.

**Table 4:** Logistic Regression Analyses of Individual-Level and Neighborhood-Level Factors Associated With Smoking Status in the Hawaiian Homestead Health Survey, 2020

Variable^a^	β (95% CI)	OR (95% CI)	*P* value
**Sociodemographic factors**			
Sex	−0.48 (−1.16 to 0.21)	0.62 (0.32 to 1.23)	.17
Age	0.01 (−0.01 to 0.03)	1.01 (0.99 to 1.03)	.44
Education level^b^			
Less than high school graduate	−0.72 (−2.14 to 0.70)	0.49 (0.12 to 2.02)	.32
High school graduate or General Educational Development certificate	−0.89 (−2.37 to 0.59)	0.41 (0.09 to 1.80)	.24
Some college or technical school	−1.27 (−2.72 to 0.18)	0.28 (0.07 to 1.20)	.09
Income	−0.00 (−0.02 to 0.01)	1.00 (0.98 to 1.01)	.58
**Individual-level factors**			
Physical activity	−0.04 (−0.27 to 0.18)	0.96 (0.76 to 1.20)	.70
Percentage of fat in diet	0.01 (−0.03 to 0.05)	1.01 (0.97 to 1.06)	.66
Body mass index	0.01 (−0.03 to 0.05)	1.01 (0.97 to 1.06)	.59
**Neighborhood-level factors**			
Walkability	−0.63 (−1.17 to −0.09)	0.53 (0.31 to 0.91)	.02
Availability of healthy foods	0.21 (−0.14 to 0.55)	1.23 (0.87 to 1.74)	.24
Safety	0.17 (−0.24 to 0.58)	1.18 (0.79 to 1.78)	.41
Social cohesion	0.23 (−0.13 to 0.60)	1.26 (0.87 to 1.82)	.21

a Details about the variables are explained in footnotes to Table 1.

b Reference group is college graduate.

The hierarchical regression analysis of individual-level CVD risk factors on physical activity included age, sex, education level, income, percentage of fat in diet, BMI, and smoking status (Table 3). The proportion of variance in physical activity accounted for in the model was significant (*R* ^2^ = 0.21, *F* = 4.46, *P* < .001). The demographic variables of sex (standardized β = −1.06, SE = 0.19, *P* < .01), age (standardized β = 0.02, SE = 0.01, *P* < .01), and education had significant, independent relationships with physical activity. The individual-level risk factor of BMI (standardized β = 0.05, SE = 0.01, *P* < .01) also had a significant relationship with physical activity. A greater BMI was associated with less frequent physical activity. None of the neighborhood-level factors had a significant relationship with physical activity. A post-hoc regression analysis, not displayed in Table 3, was conducted to examine the contribution of the 4 neighborhood-level factors as a group in explaining the variance in physical activity. The model without the neighborhood-level factors included age, sex, education level, income, percentage of fat in diet, BMI, and smoking status. This model accounted for a significant proportion of the variance in physical activity (*R* ^2^ = 0.19, *F* = 6.16, *P* < .001). When the neighborhood-level factors were added to the model, it was not significantly improved (*R* ^2^ = 0.21, *F* = 1.40, *P* = .28).

The hierarchical regression analysis of individual-level CVD risk factors on BMI included age, sex, education level, income, percentage of fat in diet, physical activity, and smoking status (Table 3). The model accounted for a significant proportion of the variance in BMI (*R* ^2^ = 0.22, *F* = 4.81, *P* < .001). The demographic variables of sex (standardized β = 3.83, SE = 1.02, *P* < .01), age (standardized β = −0.07, SE = 0.03, *P* = .04), and education had significant, independent relationships with BMI. Physical activity (standardized β = 1.48, SE = 0.34, *P* < .01) and percentage fat in diet (standardized β = 0.14, SE = 0.07, *P* = .03) both had significant, independent relationships with BMI. These results indicate that greater physical activity was associated with a lower BMI and greater percentage fat in diet was associated with a higher BMI. Of the neighborhood-level factors, only availability of healthy food had a significant, independent relationship with BMI (standardized β = 1.47, SE = 0.53, *P* = .01). However, a post-hoc regression analysis, not displayed in Table 3, was conducted to examine the contribution of the 4 neighborhood-level factors in explaining the variance in BMI. The model without these 4 factors explained 11% of the variance in BMI (*R* ^2^ = 0.11, *F* = 3.22, *P* = .001). The addition of the 4 neighborhood-level factors significantly improved the model (*R* ^2^ = 0.22, *F* change in *R* ^2^ = 7.82, *P* < .001). Because of the significant bivariate associations between the neighborhood-level factors, interaction terms were assessed. However, none were significant. The β estimates for sex, age, education, and income were similar in direction and magnitude before and after the addition of the neighborhood-level factors. The β estimate for physical activity increased in magnitude after the inclusion of the neighborhood-level factors. Additionally, we explored the interaction terms for the neighborhood-level factors and found a significant interaction in the BMI model between healthy food and safety. It indicates that in neighborhoods that are perceived as safe, the availability of healthy foods is more strongly related to BMI than in neighborhoods that are perceived of as less safe.

### Logistic regression

We performed logistic regression analysis of smoking status on sociodemographic factors, individual-level CVD risk factors, and neighborhood-level factors (Table 4). Walkability had a significant relationship to smoking status, such that as the perceived walkability of a neighborhood was worse, likelihood of ever smoking decreases (OR, 0.53; 95% CI, 0.31–0.91; *P* = .02).

## Discussion

The role of neighborhood-level factors at play in CVD risk is the subject of a growing investigation. We examined the relationship between neighborhood-level stressors on 4 established individual-level CVD risk factors, controlling for sociodemographic characteristics and lifestyle behaviors in Native Hawaiian homesteaders. Of the individual-level factors investigated, our models explained a significant proportion of variance in BMI and physical activity. Examining the contribution of the neighborhood-level stressors, both individually and collectively, only a significant relationship with BMI was found. Neighborhood-level factors, collectively, explained a significant proportion of the variance in BMI, with availability of healthy foods being of particular importance. The perception of a greater availability of healthy foods in one’s neighborhood was associated with having a lower BMI.

The literature is mixed in terms of the relationship between availability of healthy food at a neighborhood level and residents’ BMI. Similar to our study, other studies have found that a greater perceived availability of healthy food is associated with lower BMIs (26,27). Others, however, have found limited to no evidence supporting a relationship (28). A systematic review found limited evidence for an association between community food environments and BMI (29).

Despite only the availability of healthy food having a significant, independent relationship with BMI, the neighborhood-level factors as a group explained a significant portion of the variance in BMI. This finding suggests that the social (ie, social cohesion and safety) and physical (ie, walkability and availability of healthy food) environments may function together in their relationship to BMI. Our study included Native Hawaiians, a population that experiences multiple health disparities but with limited research, in the novel context of Hawaiian Homesteads. Qualified Native Hawaiians can obtain 99-year leases for $1 per year, a significant financial incentive in the state with the highest cost of living in the US (30). To qualify for leases, people must complete a lengthy application that includes providing evidence (eg, birth certificates) of at least 50% Native Hawaiian ancestry. Thus, homestead lessees are self-selecting to reside in predominantly Native Hawaiian communities. This may result in shared values among community members as well as a shared ethnicity. Additionally, residents’ ethnicity is explicitly tied to their community’s identity.

Availability of healthy foods, social cohesion, and safety had significant bivariate associations with BMI. These neighborhood-level factors were all significantly correlated with each other. Other studies have combined similar neighborhood-level factors into larger constructs, such as neighborhood environment. Mujahid et al combined walkability and availability of healthy foods to create a measure of the physical environment (27). The authors also combined safety and social cohesion (as well as aesthetics and violence, which were not included in our study) to create a measure of the social environment.

The data collection for this study occurred during the spring of 2020. Thus, it is possible that the data from this study were affected by containment and mitigation efforts associated with the COVID-19 pandemic. Research suggests that lifestyle changes during the first several months of lockdown were small (31–33). Most studies reported decreases in physical activity and increases in sedentary behaviors during their respective lockdowns across several populations (34). Social cohesion may have decreased during the lockdown (35). Utilitarian walking decreased dramatically at the beginning of the lockdown restrictions because of reductions in the needs and opportunities to walk to work, to public transport, to shop, and to other amenities (35). The decrease in walking for leisure was less pronounced in general (36). However, the COVID-19 containment and mitigation strategies may have had significant behavioral health impacts in Native Hawaiian communities (37). Increased psychosocial and financial stress increased the incidence of substance abuse and domestic violence in Native Hawaiian communities (38).

### Limitations

The results from this study should be interpreted in the context of the study’s limitations. Data presented here are cross-sectional. Thus, associations do not imply causality. The measure of neighborhood-level stressors assessed participants’ perceptions of their neighborhoods. Thus, walkability, availability of healthy foods, safety, and social cohesion may be subjected to reporting biases because they are based on self-report and perceptions. This may be particularly problematic for the walkability and availability of healthy food dimensions. For instance, some research has found a lack of agreement between perceptions of neighborhood walkability and objectively measured walkability. However, despite a lack of strong association with objective walkability, perceptions were still strongly associated with walking behavior (39,40). Other studies have found significant agreement between perceived and objective neighborhood walkability (41). Both perceived and objective neighborhood walkability were found to be associated with walking and physical activity. These findings suggest that improving perceptions of neighborhood walkability may increase physical activity (41).

### Conclusion

Among the 4 individual-level CVD risk factors examined in this study, only BMI was significantly related to neighborhood-level stressors (ie, availability of healthy food, safety, and social cohesion). The neighborhood-level factors significantly contributed to explaining BMI levels beyond sociodemographics, physical activity, and diet behaviors. Among the 4 neighborhood factors measured, availability of healthy foods had the strongest relationship with BMI after controlling for confounding variables. This suggests that greater perceived availability of healthy food in one’s neighborhood may be related to having a lower BMI.
